# Engagement of private health care facilities in TB management in Lusaka district of Zambia: lessons learned and achievements

**DOI:** 10.1186/s12889-024-18285-4

**Published:** 2024-03-14

**Authors:** Robert Hambwalula, Mary Kagujje, Innocent Mwaba, Dennis Musonda, David Singini, Lilungwe Mutti, Nsala Sanjase, Paul C. Kaumba, Luunga M. Ziko, Kevin M. Zimba, Pauline Kasese-Chanda, Monde Muyoyeta

**Affiliations:** 1 TB department, Centre of Infectious Disease Research in Zambia, Plot # 34620 Off Alick Nkhata Road, Mass Media, P.O. Box 34681, Lusaka, 10101 Zambia; 2 Lusaka District Health Office, Ministry of Health, Great East Road, Lusaka, Zambia; 3 Division of Health, United States Agency for International Development, Lusaka, Zambia

## Abstract

**Background:**

Globally, at least 3 million TB patients are missed every year. In Zambia, the TB treatment coverage increased from 66% in 2020 to 92% in 2022. Involvement of all levels of health care service delivery is critical to finding all the missing TB patients.

**Methods:**

A survey was undertaken in 15 private facilities in Lusaka district of Zambia using a structured tool administered by project team and a district health team member. Data collected during the survey was analysed and results were used to determine the type of TB services that were offered as well as barriers and enablers to TB service provision. This was followed by a set of interventions that included; training and mentorship on active case finding and systematic TB screening, increased diagnostic capacity, provision of national recording and reporting tools and provision of TB medication through linkage with the National TB program (NTP). We report findings from the baseline survey and changes in presumptive TB identification and notification following interventions.

**Results:**

Major barriers to TB service delivery were the high cost of TB diagnostic testing and treatment in facilities where services were not supported by the National TB program; the mean cost was 33 (SD 33) and 93 (SD 148) for GeneXpert testing and a full course of treatment respectively. Pre-intervention, presumptive TB identification appeared to increase monthly by 4 (*P* = 0.000, CI=[3.00–5.00]). The monthly trends of presumptive TB identification during the intervention period increased by 5.32 (*P* = 0.000, [CI 4.31–6.33. Pre-intervention, the notification of TB appeared to decrease every month by -4.0 (*P* = 0.114, CI=[-9.00-0.10]) followed by an immediate increase in notifications of 13.94 TB patients (*P* = 0.001, CI [6.51, 21.36] in the first month on intervention. The monthly trends of notification during the intervention period changed by 0.34 (*P* = 0.000 [CI 0.19–0.48]). Private facility contribution to TB notification increased from 3 to 7%.

**Conclusion:**

Engagement and inclusion of private health facilities in TB service provision through a systems strengthening approach can increase contribution to TB notification by private health facilities.

**Supplementary Information:**

The online version contains supplementary material available at 10.1186/s12889-024-18285-4.

## Introduction

Every year, at least 10 million people fall ill from TB and over 1 million people die from TB worldwide [1–3]. Only between 58%-70% of the global incident TB cases were diagnosed and put on treatment between 2020 and 2022, missing at least 3 million TB patients every year [1–3]. The Africa region accounts for about 25% of the missed TB cases globally [1–3]. Zambia, one of the 30 high TB burden countries, has been showing consistent progress in TB case identification. The TB notifications increased from 40,000 in 2020 [3] to 54,100 in 2022 [1] translating into an increase in TB treatment coverage from 66 to 92% during the corresponding period. Finding the missing TB cases is critical to meeting the End TB targets and requires the involvement of all levels of health care service delivery [4].

There are several barriers to finding the missing TB patients, among them is the limited involvement of private healthcare providers. This is an especially important barrier as private providers may account for 50% of care in urban settings in low- and middle-income countries [5]. Available evidence shows that the engagement of private care providers plays an important role in increasing TB case detection [6–10]. This is both through strengthening TB case detection strategies and reporting [11].

Zambia has 307 private health facilities which make up 9% of all healthcare facilities [12]. There is limited literature from Zambia on the engagement of private care providers in TB care. A study conducted in two public health facilities in Lusaka reported that 12% of TB patients reported that they first sought care for their symptoms from private facilities [13]. Results from the national TB prevalence survey indicate that 4% of the symptomatic undiagnosed TB patients had previously sought care for their symptoms in the private sector [14].

The Centre for Infectious Disease Research in Zambia (CIDRZ), through the United States Agency for International Development (USAID) funded, Tuberculosis Local Organization Network (TBLON) project embarked on strengthening the contribution of the private sector to TB notifications. The purpose of this paper is to share the results of targeted systems strengthening interventions to improve presumptive TB identification, TB diagnosis and notification in private health facilities.

## Methods

### Baseline assessment

A baseline assessment was undertaken in 15 out of 59 private facilities in Lusaka district between April 2020- June 2020 to determine the type of TB services offered as well as barriers and enablers to TB service provision in the private sector. The selection of private facilities for assessment was based on the following criteria: a high volume of clients (based on outpatient attendance), servicing clients from low-income communities and availability of HIV treatment services. The facilities were ranked using this criterion and the top 15 were selected for the baseline assessment and to be in phase 1 of the interventions following the baseline assessment. The above criteria were aimed at prioritizing facilities with a high burden of TB [15, 16]. The interventions were implemented in a phased approach and, by November 2023, twenty-four [24] out of 59 private facilities in Lusaka district had been included. There were no financial incentives offered and none of the facilities that were selected requested financial incentives. However, the linkage to the district health office with access to program supplied TB medication and TB diagnostic tests was offered as an incentive.

The private health facilities were assessed using a structured tool which was administered by a team comprising a TB/HIV mentor and a data associate from the project, and a monitoring and evaluation officer from the Ministry of Health (see supplementary material 1). Specifically, the appointed representative(s) of the private health facilities provided responses to the of the questions in the assessment tool. Additional information was obtained through onsite inspection of the facilities, and where TB presumptive and notification data was available, this was also collected.

### Data sources and analysis

#### Baseline assessment

Descriptive analysis was done on the baseline assessment data using simple tables that included: level of services provided, human resources capacity for TB services provision, facilitators for TB service delivery, laboratory services and recording and reporting capacity for TB service delivery. The gaps and barriers that were identified during the baseline survey were used to determine targeted interventions to strengthen TB case finding, TB treatment and linkages between the private health facilities and the district health office.

### Presumptive TB, notification and treatment outcome

Aggregated data was collected from paper source reporting and recording tools that are used in routine services in health facilities. Data that was collected included presumptive TB data, notification data, and treatment outcome data aggregated into monthly intervals. Data was entered into Facility Information Management System (FIMS), a web-based DHIS2 platform. Structured Standard Operating Procedures (SOPs) and data collection tools were designed to guide data collectors and ensure complete, accurate, and correct data entry. Rigorous data verifications were done to validate the data before reporting. Additionally, data quality audits were conducted by the project team and the Ministry of Health. Reported data was cross checked against data from the source documents and any identified variances were corrected. Private health facilities were also invited to join the quarterly TB data review meetings aimed at reviewing facility data and to identify and remove any data errors or inconsistences and promote data ownership. The data was later extracted and exported into Stata (Copyright 1985–2021 StataCorp LL StataCorp) for analysis.

For the baseline data, simple descriptive analysis was done, and results were presented as frequencies and means. For the 15 phase 1 intervention facilities, interrupted time-series analysis was undertaken using segmented linear regression. Time series analysis assumes that that the pre-intervention period trend does remains the same during the intervention period if the intervention has no impact. Two time periods were defined: Before intervention (Period 1 was April 2020 to June 2020); during intervention (period 2 from July 2020 to November 2023). The ITSA Stata command was used to conduct the interrupted time series analyses.

Further descriptive analysis was undertaken to determine the contribution of the private health facilities to Lusaka district notifications for all 24 private health facilities (including the 15 phase 1 intervention facilities) as well as the treatment success rate. Treatment success rate was defined as the proportion of TB cases registered in a given year that successfully completed treatment without bacteriological evidence of failure [17]. Utilisation of LF-LAM was also reported.

### Interventions in response to baseline survey findings

#### Orientations on TB active case finding (ACF) to increase TB notification

To increase TB case detection, orientations on TB ACF started in July 2020. The team conducting this orientation used a standardised power point presentation that was developed by the project team and the NTLP. The themes covered during the orientation included; 1) Global and local burden of TB; 2) Clinical presentation TB; 3) Systematic TB screening and diagnostic algorithms for TB in Zambia; 4) Evidence of impact of ACF; 5) Explanation of TB case detection and losses using the TB care cascade and the ‘onion’ model of case detection” and 6) Laboratory diagnosis of TB. The orientations were aimed at raising the index of suspicion of TB and introducing TB screening for all the clients that visit the private health facilities. All departments at each facility were represented during ACF orientation meetings and this gave a clear understanding of what role each department had to play in TB screening. The staff included clinicians, pharmacists, nurses, laboratory, radiology, and front office; and meetings were held during lunch break or during the weekend.

### Mentorship in TB screening

Following the initial ACF activation meeting, at least weekly mentorship visits were conducted to address knowledge gaps among healthcare providers in the private sector. Depending on need, some facilities had more than one mentorship visit in one week. Healthcare workers from private facilities were mentored on the systematic screening of TB according to Zambian guidelines, TB sample referral, TB case management, and documentation, among others. The duration of the mentorship was variable and was dependent of the facility needs. Mentorship was followed by technical and supportive supervisory visits that were conducted in collaboration with the district TB coordinator on a quarterly basis.

### Increased TB diagnostic capacity

To reduce the cost of TB diagnosis and to increase access to TB diagnostic services, private facilities were linked to public TB diagnostic facilities through a sample courier system. The district health office had an existing sample courier system that is used to move samples within the public health services; Zambia uses a hub and spoke system for access to Xpert MTB/RIF services because not every facility has an Xpert machine. Private health facilities were joined to this existing courier system and the courier of samples was provided on demand; private facilities made a phone call to the designated courier for the zone who collected the sample and had the responsibility to return the results.

In addition, urine lipoarabinomannan (LAM) test kits were supplied. The delivery of the LAM kits was accompanied by an orientation on guidelines for use of LAM as well as training of personnel on how to perform the LF-LAM test.

### Provision of reporting and recording tools and orientation on generation of TB reports

National TB recording and reporting tools which included TB presumptive registers, TB Treatment registers, TB Treatment Cards, TB identity cards, TB contact tracing registers, and TB preventive therapy (TPT) registers were supplied. This was followed by an orientation on documentation in registers and how to generate TB reports.

To facilitate notification of TB patients, the provision of reporting tools was accompanied with establishment of the private facilities as notification centres/basic management units (BMUs) or treatment centres. Working in conjunction with the district health office, the three additional BMUs were established. The rest that did not have any systems in place for reporting TB patients into the national reporting system were linked to public health facility BMUs and they became TB treatment centres. All facilities received support to strengthen their reporting systems, to ensure that reported data was accurate. Data quality audits were done every quarter in the same way they were done in the public facilities and corrective actions taken.

### Provision of TB drugs to be offered for free to TB patients

To reduce the cost of TB treatment, private facilities were linked to the District Health Office to facilitate access to free anti-TB medicine. In turn, private facilities were required to submit monthly TB notification and treatment reports to account for the distribution and usage of the medications they received. Anti-TB medicines were supplied monthly per patient diagnosed and notified until the patient completed treatment.

## Results

### Findings from baseline assessment

Of the 15 facilities assessed, 13 (87%) were hospitals and 7 (47%) were providing onsite TB diagnostic and/or treatment services (Table 1). Of the 7 providing TB services, five [5] facilities that had diagnosed a TB patient between April 2019- June 2020, 3 (60%) had notified the patients to the National TB program. There was variable access to TB recording tools with the TPT register being the most widely available in 8(53%) facilities; 7(47%) had TB treatment registers in place. Less than 40% of facilities had access to any of the adult TB, childhood TB or MDR TB guidelines. The average cost of GeneXpert and microscopy were $33 and $5 respectively with 1 of the facilities providing free services with support from the National TB program (NTP)(average cost in only fee-paying facilities were $41 and $7 respectively). The average cost of TB treatment was $93 with 4 of the facilities providing free services with support from NTP (the average cost in only fee-paying facilities was $280). Only 20% of private facilities reported existing engagement with public health facilities which included referral of TB patients to and from public facilities and utilization of TB diagnostic and treatment services available at nearby public facilities.

**Table 1:** Characteristics of the private facilities

Variable	Sub-category	Facilities (*n* = 15)
Level of care	Hospital	13 (87%)
	Clinic	2 (13%)
Type of TB services	Onsite diagnostic services without treatment services	1 (7%)
	Onsite diagnostic and treatment services	3 (20%)
	Onsite treatment services only	3 (20%)
	None	8 (53%)
Notification of diagnosed TB patients^1^		3/5 (60%)
Availability of TB recording tools	Presumptive TB register	7 (47%)
	TB treatment register	7 (47%)
	Contact tracing register	3 (20%)
	TPT register	8(53%)
Access to TB guidelines	Adult TB	5 (33%)
	Childhood TB	3 (30%)
	MDR TB	0 (0%)
Availability of trained staff on TB	Adult TB	8 (67%)
	Childhood TB	5 (42%)
	MDR TB	0 (60%)
Mean cost of TB services (SD)	Gene Xpert^2^	$ 33(33)
	Microscopy^3^	$ 5 [4]
	TB treatment^4^	$ 93(148)
Engagement with public facilities^5^	Yes	3 (20%)
	No	12 (80%)

^1^Only 5 facilities had diagnosed a TB patient between April 2019-June 2020

^2^Data from 5 facilities of which one provides free services with support from the NTP.

^3^Data from 4 facilities of which one provides free services with support from the NTP

^4^Of the 6 facilities offering treatment services, 4 had access to free anti-TB drugs from the Ministry of health

^5^Engagement includes referral of TB patients to and from public facilities and utilization of TB diagnostic and treatment services available at nearby public facilities

### Outcomes of interventions

#### Presumptive TB identification

**Fig. 1:**
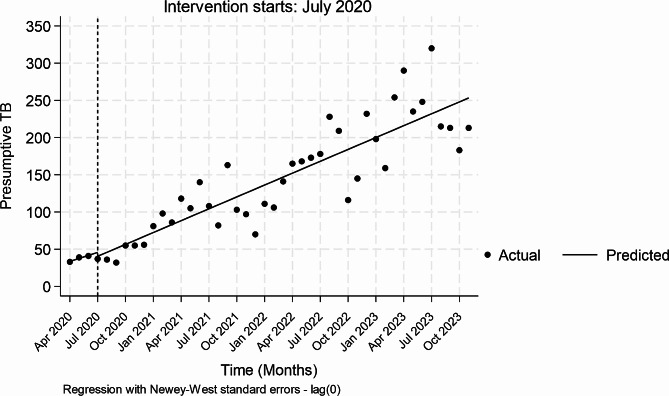
Presumptive TB patients in TBLON supported private facilities, April 2020 to October 2023

Pre-intervention, the presumptive TB identification appeared to increase monthly by 4 (*P* = 0.000, CI=[3.00–5.00]) (Table 2). During the first month of interventions, there appeared to be an insignificant reduction in presumptive TB identification of -5 patients (*P* = 0.516, CI [-21.07-10.75]). The monthly trends of presumptive TB identification relative to the pre-intervention period increased non-significantly by 1.32 (*P* = 0.068, CI-[-10.17-2.73]) whilst the trends increased monthly by 5.32 (*P* = 0.000, [CI 4.31–6.33]) during the intervention period. Figure 1 provides a visual display of the results.

**Table 2:** Single Intervention ITS analysis on Presumptive TB identification over time

_Presumptive TB	Coefficient	Newey–West std. err.	t	P > t	[95% conf. interval]
Mean monthly change pre-intervention	4.00	0.49	8.10	0.000	3.0, 5.0
Change at the first month of intervention	-5.16	7.87	-0.66	0.516	-21.1,10.7
Monthly trends relative to pre-intervention	1.32	0.70	1.88	0.068	-0.10, 2.74
Mean monthly change during-intervention	5.32	0.50	10.65	0.000	4.31, 6.33

### TB notifications

**Fig. 2:**
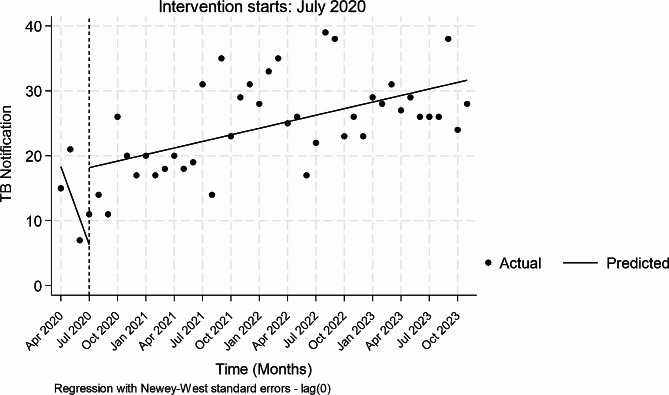
TB notifications in TBLON supported private facilities, April 2020 to October 2023

Pre-intervention, the notification of TB appeared to insignificantly decrease every month by -4.0 (*P* = 0.114, CI=[-9.00-0.10]) (Table 3). During the first month of interventions, there appeared to be a marginal increase in notifications of 12 TB patients (*P* = 0.055, CI [-0.24-9.33]. The monthly trends of notification relative to the pre-intervention period insignificantly increased by 4 (*P* = 0.087, CI-[0.66–9.33]) whilst during-intervention trends increased monthly by 0.34 (*P* = 0.000 [CI 0.19–0.48]). Figure 2 provides a visual display of the results.

**Table 3:** Single Intervention ITS analysis on TB notification changes over time

Notification	Coefficient	Newey–West std. err.	t	P > t	[95% conf. interval]
Mean monthly change pre-intervention	-4.00	2.47	-1.62	0.114	-9.00, 0 0.10
Change at the first month of intervention	11.84	5.98	1.98	0.055	-0.24, 23.91
Monthly trends relative to pre-intervention	4.34	2.47	1.75	0.087	-0.66, 9.33
Mean monthly change during-intervention	0.34	0.07	4.72	0.000	0.19–0.48

### Overall contribution of private facilities to district level notifications

**Fig. 3:**
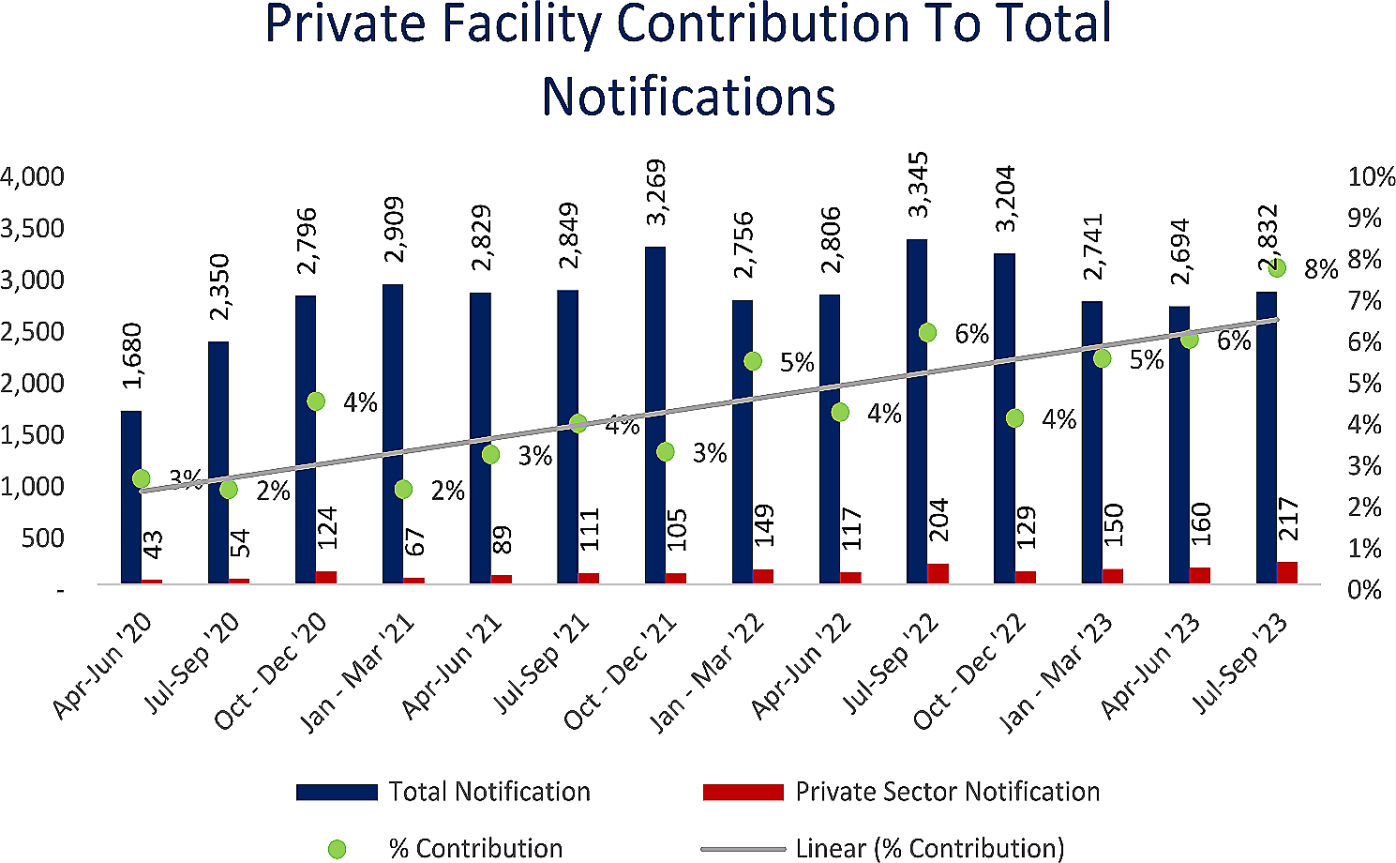
Private facility contribution to total notifications, April 2020 to September 2023

Overall, private facilities contribution to the district level notifications increased from 3 to 8% of total Lusaka district TB notifications (Fig. 3).

### LF-LAM use

Throughout the period under review 203 LF-LAM was utilized. Results from LAM use are not available.

### Treatment outcomes

For the cohort of 2020, 2021 and 2022, the treatment success was 85%, 78% and 72% respectively (Supplementary Table 1).

## Discussion

Variable access to affordable TB diagnostic and treatment services coupled with limited availability of TB recording tools and guidelines were the most common gaps and barriers identified during the baseline assessment. Facilitating provision of free TB services by the NTP, although in a limited number of facilities, and availability of staff trained in TB, although not in all facilities, were the key strengths identified during baseline assessment. Expectedly, investments in private health facility involvement in TB management resulted in increased TB diagnosis and notification as has been observed by others [18–21].

The steep decline pre-implementation is likely due to aggravation of the already existing health system challenges in TB case detection, in the private sector, by COVID-19 [22]. Similar trends were observed in national TB notification. It is worth noting that at the time of implementation of the project package of activities in the private sector, the NTP was implementing a largely similar package of activities in the public sector to minimize the impact of COVID-19 on TB notifications [23]. The change in notifications in the private sector during implementation was significantly smaller than that observed at national level. This is likely because the burden of TB among patients presenting to the private sector is low [14] but could also be because the TB systems in the private sector were less mature. It is worth noting that the gains in presumptive TB identification and TB case detection in the private sector were sustained during the implementation period. This is because the project used a health systems strengthening approach targeting all steps of the TB cascade, from screening to notification. Inclusion of the private facilities in routine data quality audits and data review meetings could also explain these gains.

Treatment success from patients notified through the private facilities was lower than the national average of 90% in 2022. A few studies conducted in Nigeria have reported similar findings [24, 25]. However, most studies report similar or better treatment outcomes in the private sector compared to the public sector [26–29]. The poor performance compared to the national average could be attributable sub-optimal knowledge of national guidelines on TB [30], incomplete documentation in TB registers [31] and the cost of services. Additionally, private health facilities unlike public health facilities do not have defined catchment population, patients travel from many parts of the districts or even the country. This is likely to pose a challenge to treatment monitoring and recording outcomes. Since the challenges are variable from facility to facility, the interventions need to be targeted at facility level gaps to improve outcomes.

Our study strengths include: use of an approach focused on leveraging the already existing systems in the public sector which is critical to sustainability and use of an approach that is data driven and scalable. However, it also has some important weaknesses: (1) the study was conducted in a specific district in Zambia hence its findings cannot be generalized to other settings, (2) the study did not conduct a formal cost-effectiveness analysis to provide a more comprehensive picture of the economic implications of engaging private facilities in TB services and (3) use of self-reported data from representatives of private facilities which could have affected the accuracy and reliability of some of the results.

In conclusion, investing in private health facilities TB services expectedly results in an increase of the private facilities contribution to TB notification. National TB programs must keep private healthcare facilities engaged in the implementation of TB services in order to optimize TB care across the sub-population utilizing private sector services while also minimizing time to case detection and missed TB cases at national level. Utilisation of approaches that speak to needs in a given setting increases the likelihood of success of the private sector engagement. Sustainability can be achieved through institutionalization of the public-private partnerships by the NTP to facilitate a continued program of mentorship, technical supervisory and support and data monitoring, in the same way that this is done for public health facilities.

### Electronic supplementary material

Below is the link to the electronic supplementary material.

Supplementary Material 1

Supplementary Material 2

## Data Availability

Data will be made available upon reasonable request.
