# Supplementary material for: Engagement of private health care facilities in TB management in Lusaka district of Zambia: lessons learned and achievements
Source: BMC Public Health. 2024 Mar 14;24:811. doi: 10.1186/s12889-024-18285-4 (PMC10941462; doi:10.1186/s12889-024-18285-4)
Supplement: Supplementary file 1 — Supplementary Material 1 [file 12889_2024_18285_MOESM1_ESM.pdf]

TBLON

BASELINE ASSESSMENT TOOL

PROFILE			
1.	Date	DD/ MM/YY	
2.	Province		
3.	District		
4.	Facility Name		
5.	Facility type<br>(Tick all options that apply)	Public	
		Private	
		Diagnostic	
		Treatment	
		Hospital	
		Clinic	
		Health post	
		MDR/RR TB initiating facility	
		MDR/RR DOT facility	
6.	Geo coordinates		
7.	SmartCare code		
8.	Distance from DHO		
9.	Catchment population (2022)		

HUMAN RESOURCE CAPACITY FOR TB SERVICE PROVISION			
**HUMAN RESOURCE AT THE FACILITY**		NUMBER	COMMENT
10.	Medical Doctors		
11.	Medical Licentiates		
12.	Clinical Officers		
13.	Nurses		
14.	Pharmacy personnel		
15.	Laboratory personnel		
16.	Environmental Health Technologist		
17.	Data clerks/associates		
18.	Nutritionist		
19.	Social workers		
20.	General workers		
21.	Community HCWs		
22.	Psychosocial counselor		
23.	Other		
**TRAININGS AND ORIENTATIONS**		NUMBER TRAINED	POSITIONS OF STAFF TRAINED/ORIENTED
24.	Adult TB training		
25.	Pediatric TB training		
26.	MDR TB training		
27.	Community Health workers training		
28.	Orientation/training on radiological diagnosis of TB		
29.	Orientation/ training in Drug surveillance and monitoring		
30.	Orientation/training in active TB case finding		
31.	Orientation/training in TB infection control		

32.	Orientation/training in TB preventive therapy		
33.	Laboratory TB Training		

	**TB/ ART SERVICES**	AVAILABILITY	
34.	TB/ ART Integration- One stop TB/HIV clinic	[Y]	[N]
	**Infection Control Measures in place**		
35.	*An infection control committee in place*	[Y]	[N]
36.	*A written site – specific infection control (IC) plan in place*	[Y]	[N]
37.	*Designated sputum collection area*	[Y]	[N]
38.	*Open waiting space*	[Y]	[N]
39.	*Average waiting time for diagnosed TB patients at the TB clinic*		
40.	*Is health education conducted daily at the TB clinic*	[Y]	[N]
41.	*If not, how often is health education provided*	[0] never	
		[1] monthly	
		[2] every 2 weeks	
		[3] weekly	
		[4] every 2-4 days	
42.	*Are the topics for health education documented*	[Y]	[N]
43.	*If yes, what areas are covered at least every week-refer to records on health education(tick all that apply)*	Adherence to treatment	
		Sputum handling and disposal	
		Contact tracing	
		Diet	
		Keeping windows open	
		Reporting of adverse effects	
		Other (specify)	
44.	*Is health education on TB symptoms and early health seeking behavior provided at other departments (tick all that apply)*	OPD	
		ART	
		MCH	
		Nutrition	
		Other	
45.	*If yes, on average, how frequently is health education provided to all the departments TB*	1] monthly	
		[2] every 2 weeks	
		[3] weekly	
		[4] every 2-4 days	
46.	*Are MDR TB patients separated from drug susceptible TB patients*	[Y]	[N]
47.	*If yes, how are they separated*	They are seen in different clinic areas	
		They are seen at different hours of the day	
		They are seen on different days	
		Other (specify)	

48.	TB/HIV coordinating body in place (minutes of meetings)	[Y]	[N]

**ENABLERS FOR SERVICE PROVISION** *(indicate either Yes or No, and record Quantities where Yes is ticked)*				
		AVAILABILITY		Quantity
**MEDICAL SUPPLIES**				
49.	Weighing scale	[Y]	[N]	
50.	BP Machine	[Y]	[N]	
51.	Stethoscope	[Y]	[N]	
52.	Pulse oximeter	[Y]	[N]	
53.	Thermometer	[Y]	[N]	
54.	Phone	[Y]	[N]	
55.	Filling Cabinets	[Y]	[N]	
56.	Cooler boxes	[Y]	[N]	
57.	Specimen bags	[Y]	[N]	
58.	Consistent supply chain for sputum bottles	[Y]	[N]	
59.	Consistent supply chain for sharps boxes	[Y]	[N]	
60.	Consistent supply chain for gloves	[Y]	[N]	
61.	Face masks	[Y]	[N]	
62.	N95 respirators	[Y]	[N]	
63.	Handwashing equipment	[Y]	[N]	
64.	Stadiometers	[Y]	[N]	
65.	Hand wash soap	[Y]	[N]	
**NTP GUIDELINES**				
66.	Childhood TB	[Y]	[N]	
67.	TB manual	[Y]	[N]	
68.	MDR TB	[Y]	[N]	
69.	Consolidated TB guidelines	[Y]	[N]	
70.	Consolidate National HIV treatment and prevention guidelines	[Y]	[N]	
71.	TPT	[Y]	[N]	
72.	TB infection control	[Y]	[N]	
**JOB AIDS**				
73.	Childhood TB	[Y]	[N]	
74.	Adult TB	[Y]	[N]	
75.	MDR TB	[Y]	[N]	
76.	TPT	[Y]	[N]	
77.	TB infection control	[Y]	[N]	
78.	TB/COVID	[Y]	[N]	
79.	TB/HIV	[Y]	[N]	
**IEC MATERIALS**				
80.	Childhood TB	[Y]	[N]	
81.	Adult TB	[Y]	[N]	

82.	TB/HIV	[Y]	[N]	
83.	MDR TB	[Y]	[N]	
84.	TPT	[Y]	[N]	
85.	TB infection control	[Y]	[N]	
86.	TB/COVID	[Y]	[N]	

A	Electronic patient record/SmartCare Assessment	Response		Comments
87.	Does this facility have SmartCare?	[Y]	[N]	
88.	If No to question 82:<br>Any reasons why SmartCare is not in use?	Not yet Deployed ☐<br>Not Working ☐<br>Staff not Trained ☐		Reason Smartcare isn't working
89.	If Yes to question 82:<br>Is the deployed SmartCare at the facility in eFirst or eLast?	E-First ☐<br>E-Last ☐		
90.	Is SmartCare being used in the TB clinic?	[Y]	[N]	
91.	If Yes to question 85: Is it being used in elast or efirst mode	E-First ☐<br>E-Last ☐		
92.	If Yes to question 85: Are there sufficient computers in the TB clinic?	[Y]	[N]	
93.	If Yes to question 85: Who primarily enters the data?	Data Associate ☐<br>Clinician ☐<br>Treatment Supporter ☐<br>Volunteer ☐<br>Nurse ☐<br>Other ☐		
94.	If No to question 85: Any reason why?	Not yet Deployed ☐<br>Not Working ☐<br>Staff not Trained ☐		Reason Smartcare isn't working
95.	Are the SmartCare computers using the latest version of SmartCare? (to specify the version)	[Y]	[N]	
96.	Do they have anti-virus software on them that is up to date?	[Y]	[N]	
97.	What kind of network do the computers connect to?	WIFI ☐<br>Wired LAN ☐		
98.	Are the computers connected to a power backup system?	Solar ☐<br>UPS ☐<br>Inverter w/o Solar ☐<br>Generator ☐		

99.	Are the staff at the site trained in the use of SmartCare?	[Y]	[N]	
100.	If Yes: How many?			
101.	If No: How many staff need training in SmartCare			
102.	Are there any staff trained in SmartCare troubleshooting	[Y]	[N]	
103.	Does this facility have YATHU?	[Y]	[N]	
104.	Does this facility have any other patient data recording system?	[Y]	[N]	
**B**	**M&E Processes**			
	Are the following data sources are available?			Quantity
105.	Presumptive TB register	[Y]	[N]	
106.	Community Presumptive TB register	[Y]	[N]	
107.	Contact tracing forms	[Y]	[N]	
108.	Contact tracing register	[Y]	[N]	
109.	TPT register	[Y]	[N]	
110.	TB treatment cards	[Y]	[N]	
111.	TB ID cards	[Y]	[N]	
112.	TB treatment register	[Y]	[N]	
113.	Request for bacteriological examination of sputum	[Y]	[N]	
114.	Request form for culture and DST	[Y]	[N]	
115.	TB laboratory register	[Y]	[N]	
116.	MDR TB treatment card	[Y]	[N]	
117.	MDR TB treatment register	[Y]	[N]	
118.	Culture sample/results register	[Y]	[N]	
119.	TB appointment register	[Y]	[N]	
120.	Transfer forms	[Y]	[N]	
121.	Mortality audit forms (TB death review forms)	[Y]	[N]	
122.	Do you have any other registers apart from the ones listed here?	[Y]	[N]	
123.	Who is responsible for recording data in the register/data sources?	Data Associate ☐<br>Clinician ☐<br>Nurses ☐<br>Volunteer/TS ☐<br>Other ☐		
124.	What challenges are there when recording data in the registers/ data sources?			
125.	Who is responsible for compiling the reports?	Data Associate ☐<br>Clinician ☐<br>Nurses ☐<br>Volunteer ☐<br>Other ☐		
126.	Who is responsible for reviewing aggregate data before its submitted?	Data Associate ☐<br>Clinician ☐<br>Nurses ☐<br>Volunteer ☐<br>Other ☐		
127.	Who is responsible for submitting the reports?	Data Associate ☐<br>In-charge ☐		

		Clinician ☐<br>Nurses ☐<br>Volunteer ☐<br>Other ☐		
128.	Can you provide templates of reports?	[Y]	[N]	
129.	Are all the TB relevant staff trained in HMIS?	[Y]	[N]	
130.	How do you ensure that the numbers entered are accurate and complete?			
**C**	**Site has updated guidelines for reporting printed and easily accessible for reference**			
131.	Does the site have printed copies of the most updated indicator guidance maintained by the designated facility staff member?	[Y]	[N]	
132.	Does the site have a comprehensive list of fiscal year targets posted and visible at TB Department? **If yes, document facility target on TB notification**	[Y]	[N]	
133.	Does the site have job-aids of indicator definitions posted and visible at TB department?	[Y]	[N]	
134.	Does the site have the posted SOPs on steps for aggregating and reporting monthly and quarterly indicators?	[Y]	[N]	
**D**	**Site conducts data review on a regular basis**			
	Does the facility conduct data reviews on a regular basis prior to reporting?	[Y]	[N]	
135.	Is there documentation of these data reviews (i.e., meeting minutes)? (VISUALLY VERIFY)	[Y]	[N]	
136.	Do facility staff and IP-staff participate in the development of data presentations for monthly and quarterly site-level data review meetings?	[Y]	[N]	
**137.**	Are action plans developed and revisited during these meetings? (VISUALLY VERIFY)	[Y]	[N]	
138.	Does the site reconcile monthly results across different data aggregation tools (i.e., between Registers, SmartCare MER and HIA2 reports)?	[Y]	[N]	
139.	Is there documented evidence of reconciliation available at the clinic (e.g., meeting minutes, results of cross-checks, etc)?	[Y]	[N]	
140.	Is there evidence of TB data use at facility level in the past 12 months (existence of graphs, charts or periodic data review reports)?	[Y]	[N]	
141.	Does the facility conduct TB mortality reviews (TB death reviews)?	[Y]	[N]	
142.	If yes to Q141, how often is it done	Monthly ☐<br>Quarterly ☐		
143.	If yes to Q141 how is it done?			

**LAB DIAGNOSTIC SERVICES**				
**LAB EQUIPMENT AND SUPPLIES (Skip this section if not diagnostic facility)**				
144.	GeneXpert	[Y]	[N]	Microscopy

145.	No of modules	Skip if not Xpert on site		Fluorescent Microscope	[Y]	[N]
146.	No of functional modules			ZN microscope	[Y]	[N]
147.	What type of laboratory information system being used for TB in the laboratory?	[1] DISA<br>[2] eLabs<br>[3] Other, specify___________<br><br>Skip if not available on site				
148.	Does DISA transmit TB results electronically to clinicians?	Skip if not available on site				
149.	Does eLabs transmit TB results electronically to clinicians?	Skip if not available on site				
150.	Internet connectivity	[Y]	[N]			
151.	Does the facility use TB LAMP?	[Y]	[N]			
152.	Does the facility use TB LAM?	[Y]	[N]			
153.	Is the Back-up power solution available?	[Y]	[N]	[Y]	[N]	
154.	Date of last EQA for smear microscopy	DD/MM/YYYY		DD/MM/YYYY		
155.	State the results of last smear microscopy EQA					
156.	Date of the last proficiency testing for GeneXpert	DD/MM/YYYY				
157.	State the results of last GeneXpert proficiency testing (EQA)					
158.	Consistent supply of cartridges	[Y]	[N]	Consistent supply of microscopy reagents	[Y]	[N]
159.	If no, when was the last stock out?					
160.	How long did it last?					
161.	What was the cause of the stock out?	[1] laboratory didn't place an order<br>[2] laboratory placed an order late<br>[3] poor quantification by the laboratory<br>[4] order placed; district store supplied less than requested<br>[5] order placed, no supply from district store<br>[6] Reagents expired			[1] laboratory didn't place an order<br>[2] laboratory placed an order late<br>[3] poor quantification by the laboratory<br>[4] order placed; district store supplied less than requested<br>[5] order placed, no supply from district store<br>[6] Reagents expired	

**COURIER SYSTEM**		
162.	What is the frequency of courier system at the facility?	[0] Never<br>[1] Quarterly<br>[2] Monthly<br>[3] Weekly<br>[4] twice a week<br>[5] Daily<br>[6]Call on demand
163.	Is the TB courier system integrated with other programs? (e.g. HIV)	[Yes] [No]
164.	Which institution is supporting the current intra district courier system?	1] PHO<br>[2] DHO<br>[3] Facility<br>[4] IP, specify:_____________________
165.	Are the courier system packaging, transportation materials available?	[Yes] [No]
166.	What is the average turnaround time of TB results for sample courier? (Randomly sample 30 recent results)	
167.	Have you ever sent to send a sample to the culture laboratories?	[Y] [N]
168.	If yes, how easy is it (was it) to have a sputum sample transported to the culture laboratories?	[1] Very easy<br>[2] Easy<br>[3] Difficult<br>[4] Very difficult
169.	How is(was) the sample sent to the culture laboratory?<br>(tick all that apply)	[1] Through the province<br>[2] Through the district<br>[3] Through partners<br>[4] Using public means
170.	On average, how long does it (did it) take you to receive the results?	[0] Results were not received<br>[1] 2-3 months<br>[2] 3-4 months
171.	If diagnostic center, how many facilities send samples for processing?	

**RADIOLOGY (X-RAY) SERVICES**			
172.	Does your facility have an X-ray machine?	[Y]	[N]
173.	If yes what type of machine is available	analogue	digital
174.	Is the X-ray machine functional	[Y]	[N]
175.	Is there someone who knows how to read the obtained radiographic images? (tick)	A radiologist	
		A radiographer	
		A radiology technologist	
		A trained clinician (medical officer, clinical officer, nurse, other)	
176.	If there is no one able to read/interpret the radiographic images, what happens then? (tick)	Radiographs transmitted electronically to a radiologist or other experienced reader at a remote site	
		Radiographs physically transmitted to a radiologist or other experienced reader at a remote site	

		Other (specify)	
177.	Do you use any artificial intelligent system to interpret the images (e.g CAD4TB)	[Y]	[N]
178.	How frequent do you experience breakdown /machine does not work in a month	weekly	
		Every two weeks	
		One in a month	
179.	Do patient pay for X-ray services?	[Y]	[N]
180.	If patient pay, how much?	Less than K50	
		K50	
		K100	
		More Than K100	
181.	If no X-ray machine, how far is the place with X-Ray services		
182.	If patient pay, how much?	Less than K50	
		K50	
		K100	
		More Than K100	

	**PUBLIC PRIVATE PARTNERSHIP**	AVAILABILITY	
	**Public facility only**		
183.	Does the facility receive any referrals from private facilities to evaluate patients for TB?	[Y]	[N]
184.	If yes to Q183 , which facilities do you usually receive referrals from(name them)		
185.	If yes to Q183, does the facility provide feedback to the referring private facility when patient is received? (check evidence of feedback)	[Y]	[N]
186.	Does the TB treatment register have any TB cases whose entry point is private facility	[Y]	[N]
187.	If yes TO Q186, how many		
	**Private facility only**		
188.	Does the facility refer any patients to public facilities?	[Y]	[N]
189.	If yes to Q188, what are the commonest reason for referral (tick all that apply)	Sputum examination	
		Chest x-ray	
		To start treatment	
		Complications of TB	
190.	If yes to Q188, which facility do you usually refer patients to		
191.	Do you usually receive feedback from the site you refer patients to ?	[Y]	[N]
192.	Do you sometimes receive referrals for treatment from other private facilities?	[Y]	[N]
193.	If yes, what are the commonest reasons or referral (tick all that apply)	Sputum examination	
		Chest x-ray	
		To start treatment	
		Complications of TB	
194.	What is the cost of x-ray at this facility (indicate not applicable if facility has no chest x-ray)		
195.	What is the cost of a GeneXpert at this facility(indicate not applicable if facility has no GeneXpert)		

196.	What is the cost of microscopy at this facility(indicate not applicable if facility doesn't offer TB microscopy)			
197.	What is the cost of TB treatment at this facility(indicate not applicable if facility doesn't provide TB treatment)			
198.	Does the facility receive any Xpert cartridges from the Ministry of Health (indicate not applicable if facility has no GeneXpert)	[Y]	[N]	[N /A]
199.	Does the facility receive any microscopy reagents from the Ministry of Health (indicate not applicable if facility does not offer TB microscopy)	[Y]	[N]	[N /A]
200.	Does the facility receive anti-TB drugs from the Ministry of Health (indicate not applicable if facility doesn't provide TB treatment)	[Y]	[N]	[N /A]

**READINIESS FOR DECENTRALISATION OF MDR TB SERVICES (includes questions not covered in other sections)**		AVAILABILITY	
	**Skip this section if the facility is not a hospital**		
201.	Does the facility have an isolation ward	[Y]	[N]
202.	If yes to Q201, how many beds are in the isolation ward?		
203.	Does the facility has specialized treatment for chronic disease conditions<br>(tick all that apply)	[1] Hypertension<br>[2] Diabetes<br>[3] Respiratory conditions<br>[4] other (specify)	
204.	What monitoring tests for TB and TB/HIV treatment is the facility able to provide<br>(Tick all that apply)	[1] Chest x-ray<br>[2] Full Blood count<br>[3] Liver function tests<br>[4] Renal function tests and electrolytes<br>[5] Thyroid function tests<br>[6] CD4 count<br>[7] Viral load<br>[8] Electrocardiogram - ECG	
205.	Does the facility have sufficient waiting area for TB patients	[Y]	[N]
206.	Does the facility have sufficient additional space for storage of DR-TB drugs	[Y]	[N]
207.	Does the facility have electronic DR-TB register	[Y]	[N]
208.	If yes, is anyone trained on the electronic DR-TB register	[Y]	[N]

	**COMMUNITY ENGAGEMENT**	AVAILABILITY	
209.	Does the facility conduct any TB community outreach activities?	[Y]	[N]
210.	If yes to Q209, outline the activities	TB sensitization/Health education	
		Contact Tracing	
		Defaulter tracing	
		Follow up of patients (monitoring)	

		visit support groups/meetings	
		Other (specify)	
		Other (specify)	
211.	If yes to Q, who funds the TB community outreach activities?	MoH	
		CHAZ	
		MoH/GF	
		JSI	
		Right to Care	
		CITAM+	
		AFYA	
		Jhiego	
		Other (specify)	
212.	Does the facility organize periodic community TB stakeholder's meetings?	[Y]	[N]
213.	If yes to Q4, how frequently?	[1] Monthly<br>[2] Quarterly<br>[3] Every six months<br>[4] Once a year	
214.	Does the facility have Community Based Volunteers that conduct Community Based TB Care activities attached to TB department?	[Y]	[N]
215.	How many community-based volunteers selected or engaged, if any?	Nil	
		1	
		2	
		3	
		4	
		5	
		<5	
216.	Does the facility have an active neighborhood health committee	[Y]	[N]
217.	What role does the NHC play on the TB program/activities	Community sensitization	
		Monitoring of treatment	
		Compilation and submission of community reports	
		Identification and referral of presumptive	
		Contact tracing	
218.	Have the CBVs received any capacity building training?	[Y]	[N]
219.	If yes to previous question, when were they last trained?	This year	
		Last year	
		Two years ago	
		More than two years	
220.	Who trained them?	Facility	
		DHO	
		MoH/GF	
		ETB	

		CITAM+	
		CHAZ	
		AFYA	
		Jhpiego	
		Other (specify)	
221.	How long was the training?	1 day	
		2 days	
		3 days	
		5 days	
		More than 5 days	
222.	What modules or training curriculum was used?	MoH TS modules	
		WHO modules	
		Other (specify)	
		Not sure	
223.	What are some of the TB activities CBVs conduct at the facility and in the community?	Community sensitization	
		Monitoring of treatment	
		Compilation and submission of community reports	
		Identification and referral of presumptive	
		Contact tracing	
		Other (specify)	
224.	How are CBVs assigned tasks and distributed in your facility	ART	
		TB clinic	
		OPD	
		MCH	
		IPD	
		Registry	
		Other (specify)	
	What tools are available to CBVs to account/ document their contribution to the activities?	Community presumptive registers	
		Community referral forms	
		Community CBV reports	
		CBV monthly report	
		TB treatment register	
		Other (specify)	
225.	Do Community Volunteers produce any activity reports?	[Y]	[N]
226.	Do Community Volunteers receive stipends or any other incentives?	[Y]	[N]
227.	Are there any community-based groups of former TB patients?	[Y]	[N]
228.		CHAZ	

229.	What other partners support Community TB in your facility/district	MoH/GF	
		JSI	
		Right to Care	
		CITAM+	
		AFYA	
		Jhiego	
		Other (specify)	
		Nill	
230.	In your district are there any existing partnerships with community radio stations to air health Programmes?	[Y]	[N]
231.	How many community radio stations do you have in your district?	Nil	
		1	
		2	
		3	
		4	
		5	
		<5	
232.	What are some of the issues prevailing in your catchment that hinder people from accessing TB services at facilities	Distance	
		Culture beliefs	
		Gender	
		HCW attitude	
		Other (specify)	
233.	Are there specific issues (stigma, myths and misconceptions, occupational routines and social issues) concerning men and women that hinder their access to TB services. Please itemize.	Stigma	
		Myths	
		Misconceptions	
		Occupational Routines	
		Social	
		Other (specify)	
234.	What support structures exist in your catchment for TB patients	Nil	
		Private hospital	
		Chemistry/pharmacy	
		Treatment both	
		NHC	
		TB support group	
		Treatment supporters/CBVs	
		Other (specify)	
235.	Are there any active community nutritional support groups within your catchment area or district?	[Y]	[N]
236.	What kind of livelihood and income generating activities that exist in your community	Public services	
		Mining	
		Fishing	
		Farming	
		Other (specify)	
		Other (specify)	
237.	How do CBVs participate in these activities to support them financially?	Nil	
		Public services	
		Mining	

		Fishing	
		Farming	
		Other (specify)	
238.	Are there any congregate settings in your community/district that exposes people to the risk of TB	[Y]	[N]
239.	What are some of the specific behaviors can you share that increase the risk to TB in your community	Nil	
		Overcrowding	
		Mining	
		Smoking	
		beer drinking	
		Other (specify)	
		Other (specify)	
240.	What are some of the challenges do you experience in your community that affect your implementation of community TB activities.	Nil	
		Traditional healers	
		Churches	
		Social	
		Traditional leaders	
		distance	
		Few CBVs	
		Other (specify)	

Baseline Performance Trend Analysis																	
		**2021**				**January -March 2022**				**April -June 2022**				**July - September 2022**			
		F 15+	M 15+	F <15	M <15	F 15+	M 15+	F <15	M <15	F 15+	M 15+	F <15	M <15	F 15+	M 15+	F <15	M <15
**TB Case Detection**																	
241.	Facility attendance																
242.	Presumptive TB cases																
243.	TB sample submitted																
244.	TB Samples tested with Xpert																
245.	TB Samples tested with microscopy																
246.	TB Samples tested with Urine LAM																
247.	Number diagnosed with TB																
248.	Started on ATT																
**TB/HIV**																	
249.	Total notifications																
250.	Number started on Treatment(Bact+Clinically diagnosed)																
251.	Number with documented HIV status																
252.	HIV positive																
253.	No of HIV positive with ART start date																

TREATMENT OUTCOMES												
	Period of Enrolment	Number enrolled on treatment	Treatment success		Died		Treatment failed		Loss to follow-up		Unevaluated	
			F	M	F	M	F	M	F	M	F	M
254.	2020											
255.	Q1 (Jan – Mar) 2021											
256.	Q2(Apr -Jun) 2021											
257.	Q3(Jul – Sep) 2021											

**INDICATORS**		**2021**	**January - March 2022**	**April -June 2022**	**July - September 2022**
**DR TB SURVEILLANCE**					
258.	No of high risk for DR-TB (retreatment cases, smear positive at month 2 and after, develop TB after TPT, presumptive TB patients who contacts to DR-TB)				
259.	Number of high-risk for DR-TB cases with access to Xpert (with Xpert results documented)				
260.	Number of High-risk for DR-TB with evidence of request for culture/ DST (samples sent for culture and DST)				
261.	Number of High-risk for DR-TB cases with Culture/DST results				
**CONTACT TRACING**					
262.	Bacteriologically confirmed TB cases				
263.	Households screened for TB				
264.	Presumptive TB cases				
265.	Presumptive TB cases that submitted sample for evaluation				
266.	Diagnosed with TB				
267.	Started on ATT				
268.	Under 5 contacts initiated on TPT				

MDR/RR TB PATIENT CARE (skip Q196-202)) if facility is not an MDR TB initiating Centre		2021				January to March 2022				April to June 2022				July - Sep 2022				COMMENT
		M<15	F<15	M>15	F>15	M<15	F<15	M>15	F>15	M<15	F<15	M>15	F>15	M<15	F<15	M>15	F>15	
269.	No of MDR/RR TB patients detected at a facility																	
270.	No of MDR/RR TB patients started on treatment at a facility																	
271.	No of MDR/RR patients started on standardized oral longer treatment regimen																	
272.	No of MDR/RR patients started on standardized oral shorter treatment regimen																	
273.	No of MDR/RR patients started on individualized treatment regimen																	
274.	No of XDR TB patients																	
275.	Number contact traced																	
276.	No of patients on treatment with baseline DST																	
277.	Number of patients on treatment with up to date culture results (2 months before current date)																	
278.	Number of patients with documented adverse drug safety monitoring and management																	
	**MDR Outcomes**	MDR/RR- TB patient who Started treatment in 2018				MDR/RR- TB patient who Started treatment in 2019				MDR/RR- TB patient who Started 2020 (Jan to Mar, Apr – Jun)				MDR/RR- TB patient who Started 2020 (Jul to Sep)				
		M<15	F<15	M>15	F>15	M<15	F<15	M>15	F>15	M<15	F<15	M>15	F>15	M<15	F<15	M>15	F>15	
279.	No. of Patients enrolled on DR TB Treatment																	

280.	No of patients who completed treatment/cured (Treatment success)															
281.	Number of patients who died															
282.	No. of patients attending their semi-annual post treatment reviews															

Laboratory Indicators (Skip section if facility is not a diagnostic site)		2021	January to March 2022	April to June 2022	July - Sep 2022	COM MENT	2021	Jan to March 2022	April to June 2022	July - Sep 2022	COM MENT
		Xpert						Microscopy			
283.	Number of TB samples received										
284.	Number of TB samples processed										
285.	TB detected										
286.	Rifampicin resistance detected										
287.	Average Turnaround time (Days)										

	WASTE MANAGEMENT AND ENVIRONMENTAL RISK ASSEMENT			
	**Staff**	AVAILABILITY		COMMENT
288.	Have health care workers at the TB clinic been trained on waste management?	[Y]	[N]	If yes, how many have been trained
289.	Which kind of waste is generated in relation to TB service provision	[1] general<br>[2] infectious<br>[3] sharps<br>[4] chemicals<br>[5] pharmaceutical waste		
290.	Quantity of general waste produced/day (estimated, kg)			
291.	Quantity of infectious waste produced/day (estimated, kg)			
292.	Number of sharps boxes used/ day			
293.	Quantity of chemical waste produced/day (estimated, kg)			
294.	Quantity of pharmaceutical waste produced/day (estimated, kg)			
295.	Into which categories are medical waste separated? (tick all those that apply)	[0] no segregation<br>[1] general<br>[2] infectious<br>[3] sharps<br>[4] chemicals<br>[5] pharmaceutical waste		
296.	Proper segregation of waste is:	[0] non-existent<br>[1] bad (low)<br>2] insufficient<br>[3] satisfactory<br>[4] good<br>[5] excellent (high)		
297.	safe handling of waste is:	[0] non-existent<br>[1] bad (low)<br>[2] insufficient		

		[3] satisfactory<br>[4] good<br>[5] excellent (high)	
298.	what kind of specific containers do you use?	[0] no specific container;<br>[1] plastic<br>[2] metallic<br>[3] cardboard<br>[4] bag<br>[5] box<br>[6] other	
299.	For what reasons are there shortages, if any?	[0] no shortages<br>[1] budget<br>[2] logistical<br>[3] other (specify)	
300.	Do you have a specific color-coding system?	Y	N
301.	Is infectious waste container are lidded?	Y	N
302.	Do you have a specific area for medical waste storage?	Y	N
303.	Is the area only accessible for authorized persons.	Y	N
304.	Are different kind of waste stored in separated storage areas?	Y	N
305.	Is hazardous and non-hazardous waste collected and transported separately?	Y	N
306.	What kind of means do you use?	[0] open device<br>[1] closed device<br>[2] other (specify)	
307.	Do you think current practices offer enough security?	Y	N
308.	who generally transports the medical waste?	[0] the HCF<br>[1] municipal service<br>[2] private company (name?)	
309.	which kind of system is used for waste treatment?	[0] none<br>[1] open fire<br>[2] incinerator<br>[3] chem. Disinf<br>[4] other	
310.	What is the current capacity of the waste treatment system(s) in kg/day?		
311.	How often treatment is done per week		
312.	Any operation problems of the waste treatment system; if so for what reasons?	[0] none<br>[1] money<br>2] maintenance<br>[3] spare parts<br>[4] other	

313.	What do you do when it doesn't function?		
314.	Is the waste disposal system on or off-site?	[0] on-site<br>[1] off-site	
315.	which kind of disposal site is used for the HCW?	[0] none<br>[1] open dump<br>[2] sanitary landfill<br>[3] small burial pit<br>[4] other	
316.	Is the disposal area secured?	Y	N

Current partner support					
	**Partner**	Area of support (tick all areas of support)	Requirement of additional support		Specify additional support required
317.		[1] Strengthening TB lab services	Y	N	
		[2] Facility TB case finding	Y	N	
		[3] Community TB case finding	Y	N	
		[4] Increasing MDR TB case detection	Y	N	
		[5] improving MDR TB treatment outcomes	Y	N	
		[6] Public private partnership	Y	N	
		[7] Improved use of data for decision making including roll out of TB module for SmartCare	Y	N	
318.		[1] Strengthening TB lab services	Y	N	
		[2] Facility TB case finding	Y	N	
		[3] Community TB case finding	Y	N	
		[4] Increasing MDR TB case detection	Y	N	
		[5] improving MDR TB treatment outcomes	Y	N	
		[6] Public private partnership	Y	N	
		[7] Improved use of data for decision making including roll out of TB module for SmartCare	Y	N	
319.		[1] Strengthening TB lab services	Y	N	
		[2] Facility TB case finding	Y	N	
		[3] Community TB case finding	Y	N	
		[4] Increasing MDR TB case detection	Y	N	
		[5] improving MDR TB treatment outcomes	Y	N	
		[6] Public private partnership	Y	N	
		[7] Improved use of data for decision making including roll out of TB module for SmartCare	Y	N	
320.		[1] Strengthening TB lab services	Y	N	
		[2] Facility TB case finding	Y	N	
		[3] Community TB case finding	Y	N	
		[4] Increasing MDR TB case detection	Y	N	
		[5] improving MDR TB treatment outcomes	Y	N	
		[6] Public private partnership	Y	N	
		[7] Improved use of data for decision making including roll out of TB module for SmartCare	Y	N	

COMMENTS

Assessed By: …………………………………………………….	Position……………………………….. Date:…………………

Facility staff Name: …………………………………………..	Position:……………………………….. Date:………………
